# Clinical Evaluation of Sequential Transdermal Delivery of Vitamin B6, Compound Glycyrrhizin, Metronidazole, and Hyaluronic Acid Using Needle-Free Liquid Jet in Facial Seborrheic Dermatitis

**DOI:** 10.3389/fmed.2020.555824

**Published:** 2020-10-30

**Authors:** Xiaomin Zhang, Bizhu Luo, Huihui Mo, Lexi Liao, Shuai Wang, Juan Du, Qiuting Liu, Yanhua Liang

**Affiliations:** ^1^Department of Dermatology, Cosmetology and Venereology, Shenzhen Hospital, Southern Medical University, Shenzhen, China; ^2^The Third School of Clinical Medicine, Southern Medical University, Shenzhen, China

**Keywords:** needle-free, transdermal delivery, liquid, facial dermatitis, seborrheic dermatitis

## Abstract

Facial seborrheic dermatitis (FSD) is a common facial inflammatory dermatitis. Needle-free transdermal jet injection (NTJI) is a non-invasive injection of drug solution by using a high-pressure liquid injection instrument. To explore a safer, more tolerable, and convenient medical way using NTJI in the treatment of FSD, the patients were treated with vitamin B6, glycyrrhizin compound, metronidazole, and hyaluronic acid sequentially using NTJI every 2 weeks, and only those treated for more than three times were included. A VISIA facial imaging system for the evaluation of erythema, superficial lipid level, and roughness of skin surface and a CK analyzer for biophysical parameters, including the stratum corneum hydration, facial surface lipid, and trans-epidermal water loss, were applied. Erythema was significantly reduced after every treatment (weeks 2, 4, and 6; *P* < 0.05), whereas superficial lipid level was not improved significantly until week 6 (*P* < 0.05), and roughness of the skin surface was not improved significantly during the whole treatment. The stratum corneum hydration of lesional skin was significantly increased after three times of treatment (*P* < 0.05). No observable adverse effect, such as marked erythema, blistering, or atrophy, was observed. Sequential transdermal delivery of small molecular weight drugs (vitamin B6, glycyrrhizin compound, metronidazole, and hyaluronic acid) using NTJI is a safe, low-toxicity, and take-home drug-free therapy for the treatment of FSD.

## Introduction

Facial seborrheic dermatitis (FSD), characterized by variable erythematous plaques with oily-yellow desquamation, is a common chronic inflammatory skin condition of the face with a high incidence and prevalence (1–3% in the general population, 3–5% in young adults, and 40–80% in HIV-positive individuals) (1). The exact cause of this disease has not been clearly identified; however, multiple factors such as lipids, immunological, hormones, nutritional, microbial factors (especially *Malassezia furfur*), and lifestyle factors may be associated with the development of FSD. Topical corticosteroids or other anti-inflammatory ointments have been the mainstay of treatment, but their use has been limited by potential adverse effects such as irritation, telangiectasia, skin atrophy, and rebound after discontinuation (1). An alternative treatment that is more effective and less stimulus is therefore imminently needed.

Epidermal barrier dysfunction is an important mechanism of FSD, and much current treatment for FSD (e.g., topical corticosteroids) may further disrupt the epidermal barrier function. Therefore, a comprehensive approach for FSD should include not only cause-relative but also epidermal barrier repairing. Thus, a typical formulary in the treatment of FSD should include antipruritics, anti-inflammatories, and skin barrier repairing. A combination of different therapies to treat FSD may be more effective and could be related to fewer adverse reactions as compared with monotherapy (2).

Vitamin B6 has a role in assuring normal structure and function of the skin and has been used clinically in the treatment of SD for several decades (3, 4). Glycyrrhetinic acid was also recommended in the treatment of SD, and its main active metabolite is glycyrrhizin; both of them have a wide range of pharmacological actions, including anti-inflammatory, anti-allergic, antiviral, anticarcinogenic, antithrombin, and anti-immune-mediated cytotoxicity (5, 6). It has been reported that glycyrrhizin can relieve immunoglobulin E-induced allergic diseases such as dermatitis (7). Compound glycyrrhizin has been used in the clinic, reduced the activity of the T-lymphocyte subset, helped recover lymphocytes, and had satisfactory efficacy and high safety in the treatment of psoriasis vulgaris, which is also chronic skin inflammation. In addition, the clinical features of facial skin lesions in psoriasis are similar to FSD (8). Metronidazole is anti-inflammatory via inhibition of free radical species (9, 10). Hyaluronic acid is a naturally occurring, highly conserved polysaccharide (11); it can trigger built-in immune defense mechanisms and promote cytokine production. Biological interactions of hyaluronic acid include effects on cell proliferation, recognition, and locomotion, along with angiogenesis and inflammatory cell activity (12).

Transdermal delivery of drugs has many advantages over other routes of administration, including the accessibility of the skin and the large surface area available for delivery. Hypodermic needles are the most commonly used instruments for the delivery because of the skin barrier by the stratum corneum. Issues such as pain, needle-stick injuries, patient compliance, needle phobia, and contamination associated with the reuse of needles have spurred the development of new drug delivery systems. Needle-free transdermal jet injection (NTJI) is a novel liquid drug delivery technology, which uses an inert gas, such as carbon dioxide, nitrogen, or helium, as a power source for drug delivery (13). The inert gas and liquid drugs are mixed and atomized to create small droplets with enough power to deliver the drugs through the skin. The jet injection technology improves drug concentration and transdermal rate and has the advantages of no pain, no needle, and no cross-infection. It can also avoid many issues associated with oral drug delivery, such as first-pass hepatic metabolism, enzymatic digestion attack, drug hydrolysis, and degradation in acidic media, drug fluctuations, and gastrointestinal irritation (14, 15).

In this study, we retrospectively analyzed patients in whom FSD had been diagnosed and treated with NTJI by sequential delivery of a formula including vitamin B6, compound glycyrrhizin, metronidazole, and hyaluronic acid.

## Materials and Methods

### Patients

**Figure 1:**
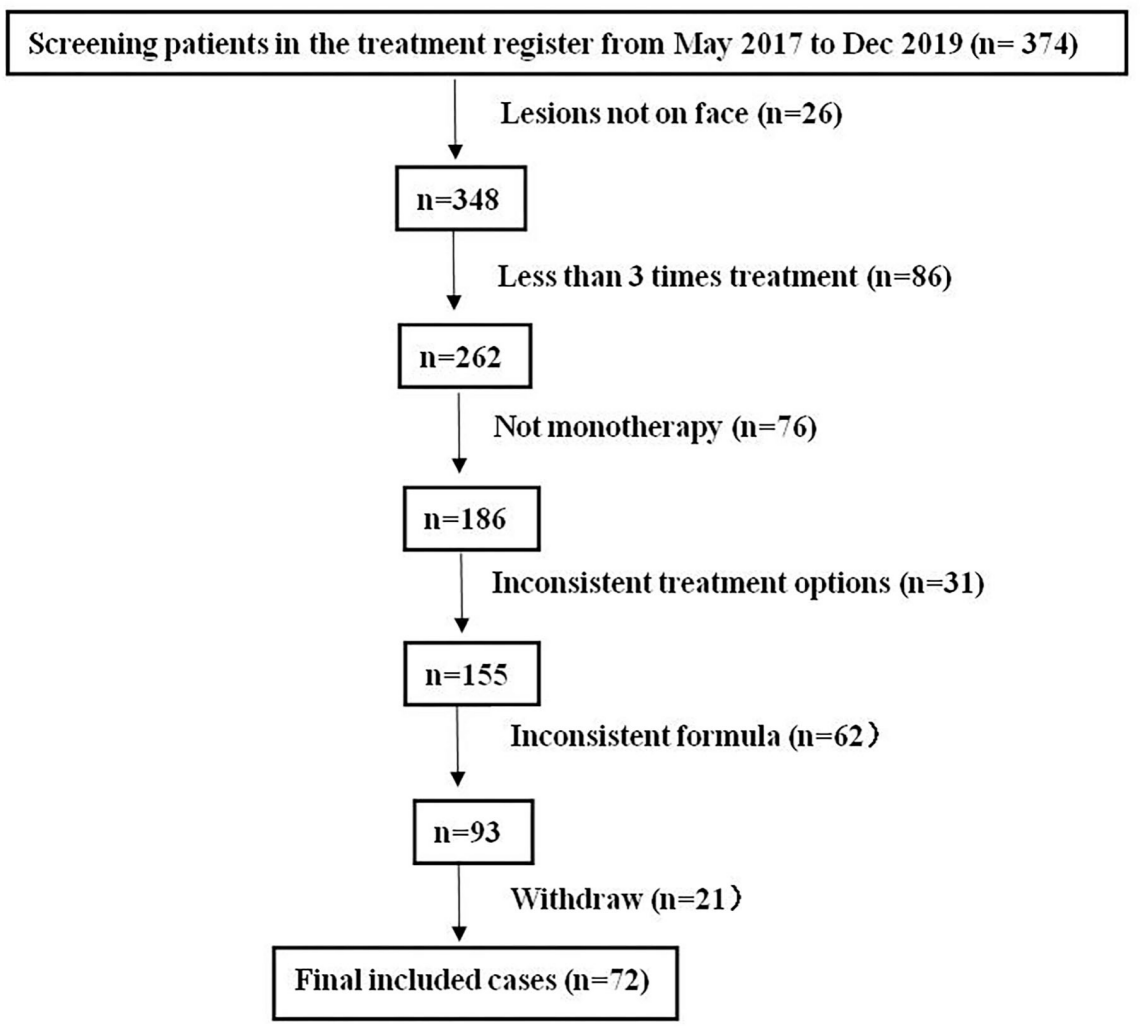
Study flow.

This study has obtained clearance from the ethics committee of Shenzhen Hospital, Southern Medical University. We retrospectively analyzed 374 patients in whom “FSD” had been diagnosed and treated with NTJI from May 2017 to December 2019. All patients had continuous symptoms of FSD, which had persisted for more than 1 month. Subjects included in the analysis were those who had undergone at least three NTJI treatments. Neither systematic medication nor other facial topical intervention during the course of the study was concurrently applied. The exclusion criteria were as follows: (1) medication of oral or topical corticosteroids, retinoids, calcineurin inhibitors, or antifungals within 2 weeks before enrollment; (2) the interval between treatments was not 2 weeks; (3) allergic to components of any drugs in this study; (4) complicated with other dermatoses in the facial area; (5) did not continue the same drug formulas; (6) with systemic disease such as diabetes, hypertension, HIV infection, and malignant tumor; (7) failure to continue follow-up; (8) having a neurological disorder or mental disorder; (9) pregnant or lactating women. The study flow is shown in Figure 1.

### Treatment Protocols

For patients with FSD, we sequentially delivered the therapeutic solutions, as 4 ml of vitamin B6, 20 ml of compound glycyrrhizin, 8 ml of metronidazole, and 6 ml of hyaluronic acid equally to the forehead, chin, and cheeks. After the whole procedure of NTJI (about 50 min), the face gets more hydration than before, making them feel more smooth and soft of their skin surface. The treatment was conducted once every 2 weeks for a total of three times. Patients were advised to avoid sun exposure during the treatment and follow-up. Compound glycyrrhizin injection (Japan Minophagen Pharmaceutical Co. Ltd., 20 ml) consists of 40-mg glycyrrhizin, 400-mg aminoacetic acid, and 20-mg cysteine hydrochloride. Vitamin B6 injection (Guangzhou Baiyunshan Tianxin Pharmaceutical Co. Ltd.) contained 50 mg/ml. Metronidazole injection (Shijiazhuang No. 4 Pharmaceutical Co. Ltd.) contained 500 mg per 100 ml. Hyaluronic acid (Wuhan Qizhi Laser Technology Co. Ltd., 20 ml) was at a concentration of 0.05% w/w. A needle-free transdermal jet injector was obtained from QIZHI Inc. (Wuhan, China). The handheld drug delivery device is powered by pressurized carbon dioxide gas. Both VISIA skin analysis imaging system (ICES-003, Canfield Scientific co, Fairfield, NJ) and CK analyzer with three detection probes (TM300, SM 815, and CM 825, Courage-Khazaka, Cologne, Germany) were applied to evaluate the efficacy.

### Assessment

#### Objective Evaluation by Both VISIA and CK Analyzer

Clinical photographs were taken on pretreatment and 2 weeks after each treatment. The effectiveness evaluation was performed by using the VISIA skin analysis imaging system. All measurements were done in the same area and location by the same physician assistant. The mean values were then calculated from these readings. The absolute scores, red area present for the extent of the erythema, porphyrins for superficial lipid level, and pores for the roughness of skin surface, which were generated by the analyzer system automatically, were used as the objective quantitative standard. The absolute score is proportional to the skin characteristics, that is, the higher the absolute score is, the more serious the symptom is. The biophysical parameters were obtained by using the CK analyzer, including facial surface lipid, trans-epidermal water loss (TEWL), and stratum corneum hydration.

#### Subjective Evaluation by Investigator Global Assessment and Patient's Self-Assessment

Investigator Global Assessment (IGA) score (11) and the patient's self-assessment were considered subjective assessments. The IGA score evaluated scale/flaking, erythema, the grade of seborrhea, and pruritus. The outcome of the treatments was evaluated by two blinded dermatologists (unaware of the times of treatment) according to patients' images. The severity of the itch was based on the description of the medical records. For each item, a 4-grade scale was used (0 = absent, 1 = mild, 2 = moderate, 3 = severe) (11).

Patients' satisfaction with the results was collected at the last follow-up. Improvement of erythematous plaques and seborrhea was scored as follows: 0, worsen; 1, no change; 2, mild (lesion clearance < 25%); 3, moderate improvement (lesion clearance 25–50%); 4, good (lesion clearance 50–75%); and 5, significant (lesion clearance > 75%) (16).

### Recurrence and Safety Evaluation

Either worsening of the erythema, scale/flaking, or enlargement of the lesion was considered a recurrence. The total recurrence rate was determined as the percentage of recurrence patients with any symptoms mentioned earlier. Adverse effects such as hypopigmentation, desquamation, edema, and erythema were recorded throughout the entire study.

### Statistical Analysis

The results were denoted by mean ± SD. SPSS 23.0 (SPSS Inc., Chicago, IL, USA) and GraphPad 7.0 were used for the statistical and graphing analysis. A coupled *t-*test was used to compare the results before and after treatments (baseline; weeks 2, 4, and 6). Chi-square was performed to evaluate patients' subjective satisfaction, and a non-parametric Wilcoxon test was used to analyze the IGA score. All P-values were bilateral, and a *P* < 0.05 or < 0.001 was defined as statistical significance.

## Results

### Baseline Data

After layers of screening per the inclusion and exclusion criteria, 72 subjects (54 females, 18 males) of age 26.84 ± 8.27 years with disease duration of 7.12 ± 2.55 months were included in the study after three times treatment of NTJI. The youngest patient in the study was 16 years of age, and the oldest was 48.

### Rapid and Continuous Improvements of Detective Index Evaluated by VISIA and CK

**Figure 2:**
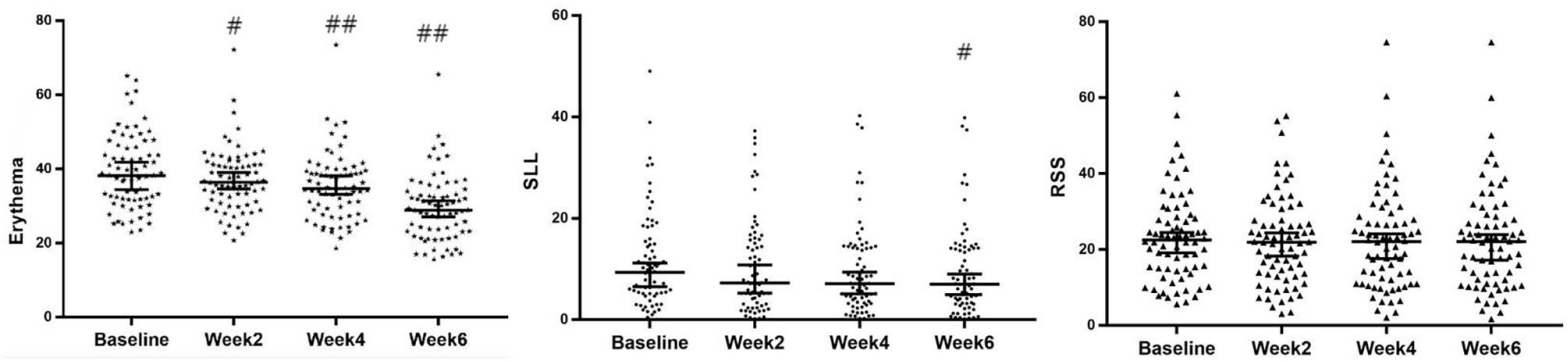
Evolution of erythema, SLL, and RSS of VISIA at baseline and 2, 4 and 6 weeks after treatment (median with 95%CI). #*P* < 0.05; ##*P* < 0.001. SLL, superficial lipid level surface; RSS, roughness of skin.

**Figure 3:**
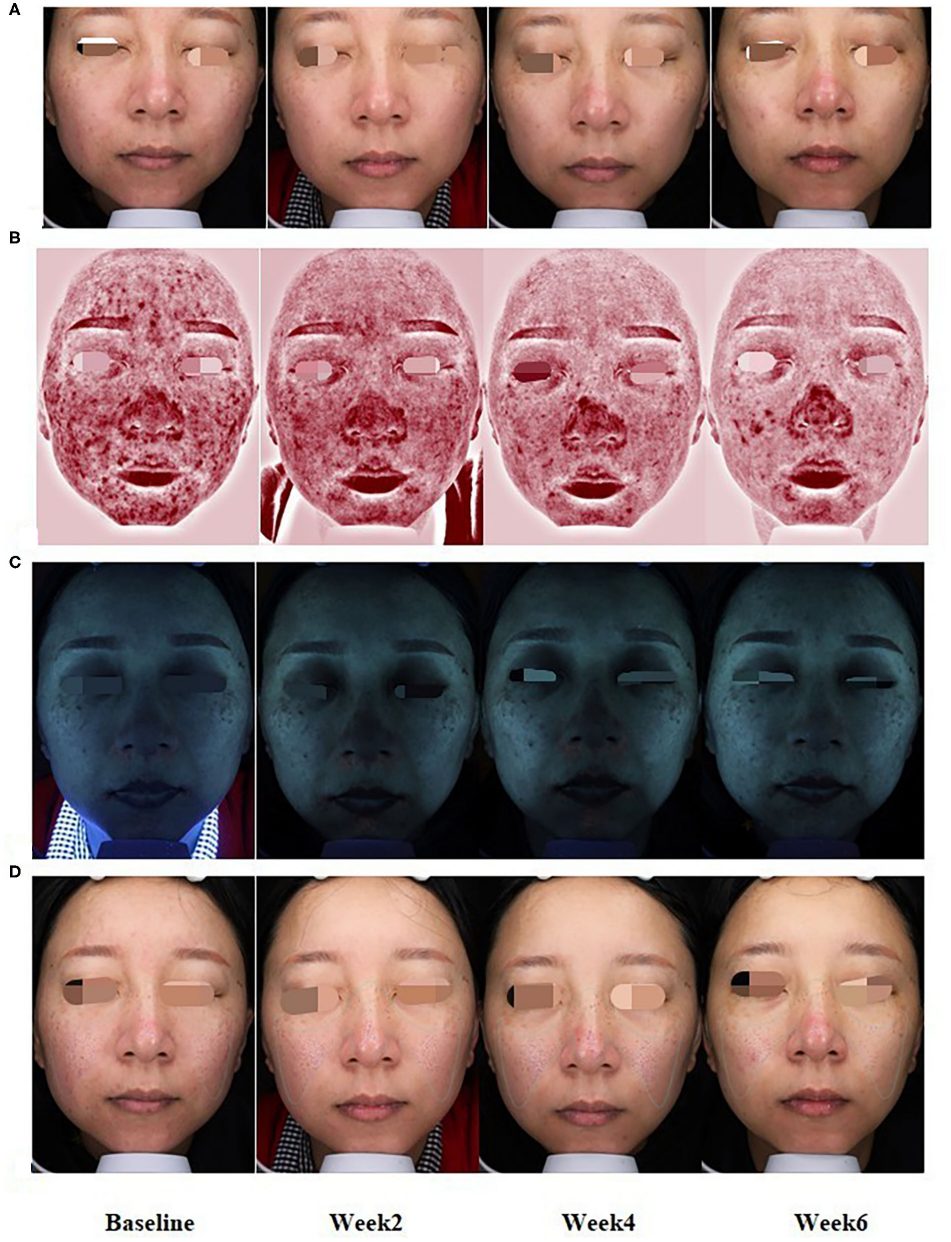
At baseline and weeks 2, 4, and 6 of treatment, representative clinical photographs of FSD patients with normal **(A)**, erythema **(B)**, lipid levels **(C)**, and skin roughness **(D)** patterns by VISIA.

In comparison with baseline data obtained before the first treatment (week 0), erythema was decreased significantly after every treatment (weeks 2, 4, and 6; *P* < 0.001), whereas the superficial lipid level was improved significantly in week 6 (*P* < 0.05) (Figures 2, 3), revealing that this treatment provided significant effect on improving the symptoms of erythema and skin sebum of FSD. However, there was no significant difference in the roughness of the skin surface in weeks 2, 4, and 6.

The mean change values of the biophysical parameter from week 0 to weeks 2, 4, and 6 are shown in Table 1. The mean of stratum corneum hydration of lesional skin in FSD patients was significantly higher after three treatments (*P* < 0.05). The facial surface lipid and TEWL scores were also comparable after three treatments, and they were improved gradually from baseline to week 6. However, the difference was not statistically significant.

**Table 1:** Biophysical parameter from baseline and after 2, 4, and 6 weeks of treatment (mean ± SD).

	**SCH**	**FSL**	**TEWL**
Week 0	43.52 ± 11.41	131.4 ± 83.22	20.07 ± 9.06
Week 2	47.27 ± 7.57	125.65 ± 77.23	18.46 ± 5.22
Week 4	48.51 ± 11.90	118.65 ± 82.60	18.05 ± 5.60
Week 6	51.88 ± 10.19*	107.25 ± 61.32	16.44 ± 6.03

SCH, stratum corneum hydration; FSL, facial surface lipid; TEWL, trans-epidermal water loss. *P < 0.05.

### Double Confirmed Therapeutic Efficacy by Investigator Global Assessment and Patients' Self-Assessment

**Figure 4:**
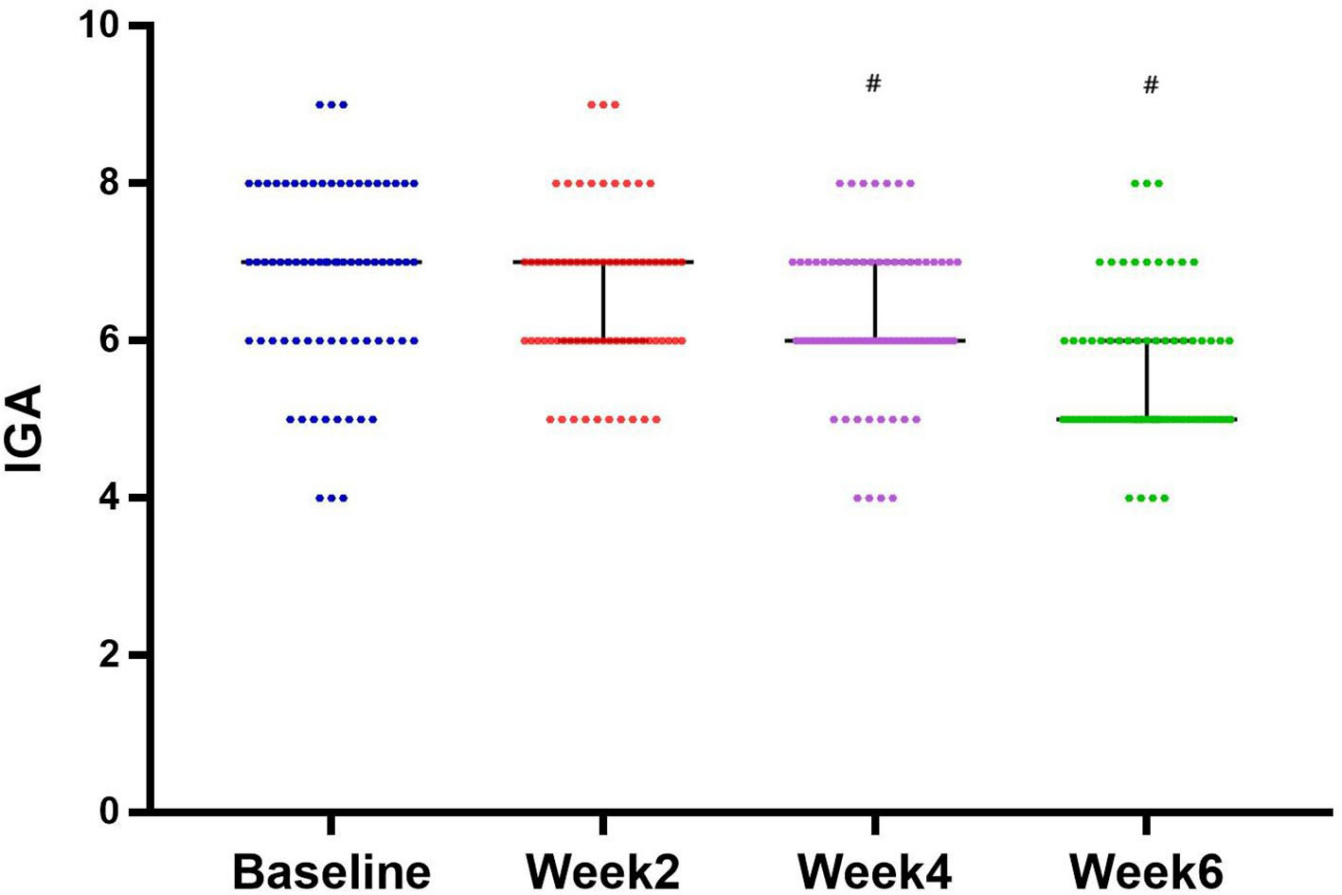
Evolution of IGA score from baseline and after 2, 4, and 6 weeks of treatment (median with 95% CI). #*P* < 0.05.

At week 0, the mean score of IGA was 6.79 ± 1.20. The treatment of NTJI reduced IGA scores significantly to 6.28 ± 0.98 at week 4 and 5.58 ± 0.93 at week 6 (*P* < 0.05). In patients' self-evaluations, 22 (31%) patients assessed the result as remarkable, 31 (43%) rated their improvement as good, 12 (17%) rated their improvement as moderate, and 7 (9%) reported poor improvement (Figure 4).

### Partial Mild Recurrence and Bit Uncomfortable Effects

At the 4-week follow-up visit, 40 patients had a recurrence of facial surface lipid, with a partial recurrence rate of 56%. No worsening of erythema or scale/flaking or itch was observed compared with that before treatment.

Adverse events were minimal in our study. Only eight patients complained of increased itching during the process of injection, and it was relieved within 3–5 min after being cold spraying with normal saline for 15 min. All patients tolerated the treatment well. No serious adverse effects such as atrophy, blistering, or obvious erythema, nor systemic symptoms were observed.

## Discussion

The diagnosis of FSD is usually made on the basis of its clinical features. The etiology remains unknown. However, *M. furfur, Staphylococcus aureus*, skin sebum levels, epidermal barrier disturbances, depression, autonomic dysfunction, and immunologic mechanisms have been described as contributing factors (5). Thus, a typically recommended prescription is a combination that should include antifungals, keratolytics, antipruritics, and anti-inflammatories (2). In general, it is complicated to decide which of these medicines should be included, and none of these four was targeting on improving skin barrier function or restoring the skin's surface lipid composite. An alternative treatment that is effective and tolerable is therefore imminently needed. NTJI technology is a user-friendly delivery method that can increase compliance in patients with FSD.

Basing on the clinical observation that patients with FSD present with excessive lipid secretion, erythema itching, and skin barrier disturbances, we sequentially delivered vitamin B6, compound glycyrrhizin, metronidazole, and hyaluronic acid to the whole face every 2 weeks for three times. Vitamin B6 is a water-soluble vitamin, which is the biologically active form and has been used clinically in the treatment of FSD for several decades. It is known that *M. furfur* plays an important role in the cause of FSD. *Malassezia* yeasts degrade sebum, releasing irritating unsaturated fatty acids and the resultant inflammation (17), whereas the latter could cause a tissue-specific depletion of vitamin B6 (18). In addition, vitamin B6 has a crucial role in the metabolism of fatty acids, lipid metabolism, and lipid peroxidation; it can reduce lipid accumulation (19). During the last 10 years, there were increasing reports implicating vitamin B6 in inflammation and inflammation-related chronic illnesses (20); it was recognized as an antioxidant and anti-inflammatory (21). Therefore, in addition to repairing the function of damaged skin, topical delivery of vitamin B6 can inhibit lipid accumulation of FSD patients.

It was found that hyaluronic acid can prevent TEWL (22), thereby improving the moisture of the skin. Draelos (23) demonstrated that a prescription hyaluronic acid-based foam device offers an aesthetic formulation with excellent efficacy in patients requiring an environment for barrier repair with mild to moderate atopic dermatitis. Both the glycyrrhizin compound and metronidazole have an anti-inflammatory effect. Due to the similar pharmacological efficacy to corticosteroids but with milder adverse effects (24), compound glycyrrhizin has been widely utilized in several dermatologic disorders for many years (6, 8). Metronidazole is an antiprotozoal and imidazole-derived antibacterial agent; it also has immunomodulatory effects on leukocyte chemotaxis and suppresses cell-mediated immunity (25), which makes metronidazole an effective therapy option for FSD.

Due to the barrier properties of the skin, transport is limited to small molecules, and it is difficult to deliver larger molecules (>500 Da) via passive transport caused by diffusion alone. In contrast, NTJI is an active transdermaldelivery that can improve the delivery of drugs of differing lipophilicity and molecular weight, including proteins, peptides, and oligonucleotides (26). NTJI is a pressure- and dose-controlled system, which functions by a compressed gas-powered device that forces liquid drug to pass at high speed through the small orifice that is close to the skin. An ultrafine, high-pressure fluid is produced that resembled the wide-fire pattern of a shotgun, so it can quickly distribute the materials and spread throughout the tissues. Therefore, it can speed up the absorption rate of the drugs and cause the drug to take effect within a shorter time (13). It was proved that although the NTJI used only lightweight atomized molecules to deliver the drug, it still provided enough power to penetrate deep into the skin layers (≈136.44 ± 10.90 μm) (13). The system has previously been used to deliver the active ingredient into a full-thickness excision wound in a diabetic mouse (27). The results show that the medication delivered by the system did not aggravate the severity of the open wound surface but increased the penetration depth and absorption of the active ingredients on the wound surface, which was conducive to wound healing. This may indicate that the micropores or microchannels produced by the system are minimal (13).

It was reported that seborrheic dermatitis is often caused by mental stress. Stress often indicates a poor prognosis, and depression is more common in patients with facial involvement. Anxiety is an aggravating factor, too (28). NTJI has the role of sedation when the cool and atomized small droplets spray on the face; patients feel calm and peaceful. Transient pruritus is the most common adverse event, and adverse events are rare and never result in systemic toxicity. Therefore, NTJI is a relatively simple procedure that is cost-effective and well-tolerated and offers both therapeutic and cosmetic benefits.

This was a retrospective study where all subjects were placed in a single cohort following the same fixed treatment strategy. The result of this study showed that delivering the comprehensive treatment package using NTJI once every 2 weeks at a total of three times could help improve the erythema and surface lipid of FSD. The one index that showed continued improvement was erythema, which is the worst facial lesion on the face impacting the patient's cosmetic appearance and confidence. The IGA and patient's satisfaction were remarkable, too.

Nevertheless, this was a retrospective study; the selection of individuals is not random, which may cause selection bias. Also, constrained by the scope of application of intervention measures, the research objects selected in this paper may not be representative of the experimental results. However, both subjective and objective assessments were used to evaluate the therapeutic effect to reduce bias. A randomized, double-blind clinical trial is needed to warrant the result of this study.

Overall, sequential transdermal delivery of vitamin B6, compound glycyrrhizin, metronidazole, and hyaluronic acid by NTJI is a rapid, effective, convenient, and safe option for the treatment of FSD. Its compliance and tolerance were excellent.

## Limitations

This was a retrospective study. A prospective study with control groups is needed to warrant the result of this study.

## Data Availability Statement

All datasets generated for this study are included in the article/supplementary material.

## Ethics Statement

The studies involving human participants were reviewed and approved by the ethics committee of Shenzhen Hospital, Southern Medical University. Written informed consent to participate in this study was provided by the participants' legal guardian/next of kin. Written informed consent was obtained from the individual(s), and minor(s)' legal guardian/next of kin, for the publication of any potentially identifiable images or data included in this article.

## Author Contributions

XZ was responsible for part of data analysis and article writing. BL was responsible for data collection. HM and LL were responsible for literature search. JD, SW, and QL were responsible for data acquisition and statistical analysis. YL was responsible for design and review of the study. All authors contributed to the article and approved the submitted version.

## Conflict of Interest

The authors declare that the research was conducted in the absence of any commercial or financial relationships that could be construed as a potential conflict of interest.
